# Task‐based evaluation of axial and multiplanar reconstructions in computed tomography: A volumetric analysis of spatial resolution, noise, and detectability

**DOI:** 10.1002/acm2.70760

**Published:** 2026-08-25

**Authors:** Pascal Monnin, Anaïs Viry, Fabio Becce, Damien Racine

**Affiliations:** ^1^ Institute of Radiation Physics (IRA) Lausanne University Hospital (CHUV) and University of Lausanne (UNIL) Lausanne Switzerland; ^2^ Department of Diagnostic and Interventional Radiology Lausanne University Hospital (CHUV) and University of Lausanne (UNIL) Lausanne Switzerland

**Keywords:** 3D image quality, computed tomography, detectability, multiplanar reconstruction

## Abstract

**Background:**

Multiplanar reconstructions (MPRs) are an essential clinical tool for interpreting complex 3D anatomy, offering improved diagnostic accuracy in computed tomography (CT). However, image quality can be impaired by the inappropriate use of reconstruction parameters for primary axial slices.

**Purpose:**

This study aimed to characterize the fundamental aspects of image quality of axial CT slices and MPRs associated with different reconstruction parameters for three recent CT systems and to propose optimal reconstruction parameters for MPRs.

**Methods:**

A dedicated cubic test‐object was designed to assess spatial resolution and noise in the primary axial slices, and in the coronal and sagittal MPRs. Two inserts with contrast levels of 120 and 1000 HU were used to measure the task‐based transfer function (TTF) in all three planes. Three‐dimensional noise power spectra (NPS) were measured in the homogeneous water volume of the cubic phantom. The TTF and NPS were used in the non‐prewhitening observer model with eye filter (NPWE) to calculate the detectability index of spheric objects with diameters from 0.5 to 5.0 mm. Three different kernels and slice thicknesses from 0.6 to 3.0 mm were tested on three recent CT systems using common iterative reconstruction algorithms.

**Results:**

MPRs are generated by reslicing axial CT images rather than by direct reconstruction from raw data. The spatial resolution was significantly lower in MPRs than in axial images, especially in the longitudinal direction when thick axial slices were used. High‐resolution and edge‐enhancing kernels produced MPRs with highly anisotropic image quality and lower detectability, while standard smooth kernels provided higher isotropy and detectability.

**Conclusion:**

MPR image quality is typically inferior to axial images and deteriorates further when derived from thicker axial slices. Therefore, appropriate selection of the reconstruction kernel, pixel size and slice thickness are essential to maintain diagnostic image quality.

## 1 INTRODUCTION

The task‐based transfer function (TTF) and noise power spectra (NPS) are well‐established metrics for image quality assessment in computed tomography (CT).^1^, ^2^, ^3^, ^4^, ^5^ Several works^6^, ^7^, ^8^ have provided a comprehensive overview of the task‐based approach methods developed for the performance evaluation of CT images, and task‐based image quality of axial CT slices has been extensively studied across various acquisition and reconstruction parameters, particularly using iterative reconstructions (IR) and deep‐learning reconstruction (DLR) techniques for various clinical tasks.^9^, ^10^, ^11^, ^12^, ^13^ Recent advances in CT hardware and software, including the developments of IR and DLR algorithms, have drastically reduced noise and artifacts while enhancing spatial resolution.^14^, ^15^, ^16^, ^17^, ^18^, ^19^ Combined with faster reconstruction speeds, it is possible to routinely consider multiplanar reconstructions (MPRs) in clinical practice.^20^, ^21^, ^22^, ^23^

Despite the growing use of coronal and sagittal images in clinical practice, little attention has been paid to the objective analysis of the image quality of MPRs, this having been evaluated and reported almost exclusively for axial slices.^11^, ^24^, ^25^, ^26^, ^27^ The spatial resolution in the longitudinal z‐direction has been evaluated using a straight edge or wire.^8^, ^28^, ^29^ However, these methods cannot evaluate the spatial resolution in an arbitrary direction. Bead or multi‐wire phantoms have therefore been developed to acquire 3D point spread function (PSF) then used to compute TTF in arbitrary directions, though this was often focused only on direct axial versus z‐axis.^30^, ^31^ The spatial resolution of MPRs characterized in specific directions using a bead has shown a simple relationship between the in‐plane and longitudinal spatial resolutions, but without a full characterization of their anisotropic spatial resolution.^32^

While the image quality of MPRs is generally accepted to be good, studies have highlighted that their diagnostic performance is highly dependent on slice thickness and image reconstruction algorithms.^33^, ^34^, ^35^, ^36^, ^37^ Accurate evaluation of 3D image quality is therefore important when considering the effects of reconstruction parameters. MPRs do not have the same resolution as axial slices and may exhibit artifacts.^38^, ^39^, ^40^ More specifically, axial and longitudinal directions do not have the same spatial resolution, and noise varies in magnitude and texture, resulting in anisotropic MPR image quality.^41^, ^42^ Therefore, proper evaluation of MPRs requires 3D image quality measurements in the CT volume to fully understand the scanner performance.

In this work, we present a new method to characterize image quality in the three orthogonal planes of the reconstructed CT volume. The method includes spatial resolution assessment, noise analysis, and detectability estimation using a Fourier domain model observer. We aimed to highlight the differences in image quality between the reconstructed planes and propose optimal reconstruction settings for primary axial slices. This quantitative study evaluates the technical capabilities and performance metrics of facilities, explicitly excluding the analysis of clinical diagnosis impact or patient outcomes.

## 2 METHODS

### 2.1 Data acquisition

Three CT systems were investigated in this study: a Canon Acquilion One Genesis (Canon Medical Systems, Ōtawara, Japan), a GE Revolution Apex (GE Healthcare, Milwaukee, WI, USA) and a Siemens Somatom Force (Siemens Healthineers, Forchheim, Germany). A Cartesian coordinate system was attached to the CT systems, where *x* refers to the left‐right (horizontal) direction, *y* to the anterior‐posterior (vertical) direction, and *z* to the head‐foot (longitudinal) direction aligned with the rotation axis of the scanner. Accordingly, the CT slices *xy*, *xz*, and *yz* thus correspond to the axial, coronal and sagittal planes, respectively.

**FIGURE 1:**
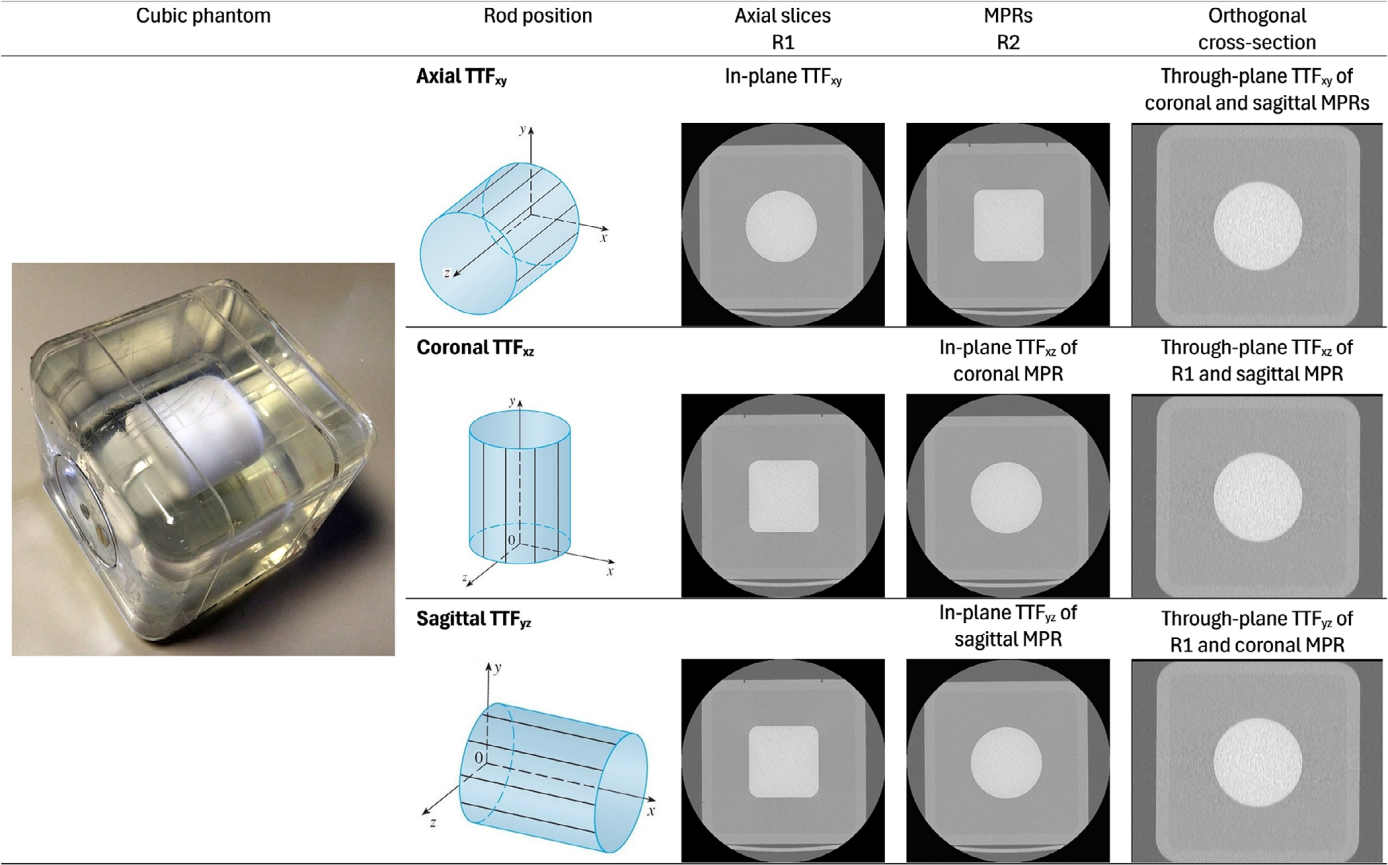
Cubic water phantom with one of the removal central rods used to calculate the TTFs and geometrical positions of the rod used for the in‐plane and through‐plane TTF measurements of R1 (axial) images and R2 (MPRs).

A dedicated water‐filled cubic phantom (Figure 1), 160 mm per side with notched edges to avoid streak artifacts, was scanned to determine the task transfer function (TTF) and the noise power spectrum (NPS). The phantom was positioned at the CT isocenter using laser alignment. A removable central rod (70 mm diameter, 70 mm length) with sharp edges was inserted at the center of the phantom to measure the TTF. The phantom was used in three configurations:

1. With a central polytetrafluoroethylene (PTFE or Teflon®) rod, CT number at 120 kVp ≈ 1000 HU simulating cortical bone, used with sharp, high‐resolution (HR) and edge‐enhancing (EE) kernels
2. With a central polymethylmethacrylate (PMMA or Plexiglas®) rod, CT number at 120 kVp ≈ 120 HU simulating dense soft tissue (e.g. cartilage or tendons), used with a soft‐tissue (ST) kernel
3. In the absence of the central rod, the homogenous water volume was used to assess the NPS.

All acquisitions were performed in helical mode with a tube voltage at 120 kVp, a pitch the closest to 1.0 and an effective tube current‐time product at 75 mAs. The detailed acquisition parameters are available in Table 1. Primary reconstructions of axial slices, denoted R1, were performed using IR algorithms available: AIDR 3D for the Canon, ASIR‐V at level 50 for the GE and ADMIRE at strength 3 for the Siemens. A reconstructed FOV of 200 mm with a pixel size of 0.391 mm (matrix 512 × 512) was used. An additional axial series was reconstructed with a doubled FOV (400 mm) and pixel size (0.781 mm). Three axial slice thicknesses were tested: submillimeter (S) between 0.5 and 0.625 mm, thin (T) between 1.0 and 1.5 mm and medium (M) between 2.0 and 3.0 mm. For each thickness, the R1 (axial) slices were reconstructed without (0 %) overlaps, and when possible, additionally with 50% and 75% overlaps. Secondary reconstructions of coronal and sagittal MPRs, denoted R2, were performed using the manufacturer's console/reconstruction software, without additional post‐processing, with a pixel size of 0.391 mm and a slice thickness between 1.0 and 1.5 mm with 50% overlap. The detailed reconstruction parameters used for the R1 (axial) slices and R2 (MPRs) are reported in Table 1.

**TABLE 1:** Acquisition and reconstruction parameters for axial slices (R1) and for coronal and sagittal MPRs (R2) (S = submillimeter, T = thin, M = medium slices).

	Canon Aquilion One Genesis	GE Revolution Apex	Siemens Somatom Force
Acquisition parameters			
Tube potential [kV]	120	120	120
Current‐time product [mAs]	75	75	75
Rotation time [s]	0.5	1.0	0.5
Pitch factor	1.0	0.984	1.0
Collimation [mm]	80 × 0.5	64 × 0.625	48 × 1.2

R1 reconstruction parameters			
Algorithm	AIDR 3D	ASIR‐V 50	ADMIRE 3
FOV [mm]	200 / 400	200 / 400	200 / 400
Pixel size [mm]	0.391 / 0.781	0.391 / 0.781	0.391 / 0.781
Kernels HR / EE / ST	FC51 / FC30 / FC18	Bone+ / Lung / STD	Br64 / Bl57 / Bf44
Slice thickness S / T / M [mm]	0.5 / 1.0 / 2.0	0.625 / 1.25 / 2.5	0.6 / 1.5 / 3.0

R2 reconstruction parameters			
FOV [mm]	200	200	200
Pixel size [mm]	0.391	0.391	0.391
Slice thickness [mm]	1.0	1.2	1.5
Slice interval [mm]	0.50	0.60	0.75

### 2.2 Task transfer function (TTF)

The cubic phantom was scanned successively with the central rod aligned along the three orthogonal axes (*x*, *y*, and *z*) of the CT systems (Figure 1) for a comprehensive assessment of both in‐plane and through‐plane resolutions of the R1 (axial) and R2 (coronal and sagittal MPRs) reconstructions. Additional orthogonal cross‐sections of the R1 and R2 stacks of images were required to measure through‐plane resolution. These cross‐sections were produced with independent software (ImageJ) to avoid voxel interpolation and thereby preserve the volumetric spatial resolution of the R1 and R2 volumes.

To accurately evaluate 3D spatial resolution of each reconstruction, two types of 2D TTFs were calculated: *in‐plane* TTFs, measured directly on R1 or R2 images, and *through‐plane* TTFs, measured on orthogonal cross‐sections of the R1 and R2 image stacks. The 2D in‐plane and through‐plane TTFs were calculated on the images where the rod appeared circular, i.e. with the central rod aligned along the z‐axis for TTF_xy_,, along the y‐axis for TTF_xz_, and along the x‐axis for TTF_yz_, as shown in Figure 1.

2D TTFs were calculated via a radial sharp edge method,^41^, ^42^ plotting the HU (Hounsfield Unit) values in polar coordinates around the rod center in square ROIs of 100 × 100 mm, perpendicularly to the rod edges, to obtain radial edge spread functions (ESFs). The rod was divided into 72 angular sections of 10° with a 5° angular pitch, to calculate radial ESFs per sector. The radial ESFs were then derived in their radial direction and Fourier‐transformed to give radial TTFs. The 72 radial TTFs were interpolated over 360° to give a 2D TTF,^36^ normalized to 1.0 at the zero frequency. Angular averages of 2D in‐plane TTFs gave mean radial 1D TTFs. The 1D inter‐slice TTFs were obtained by integration of the 2D through‐plane TTFs over the frequency bandwidth in the axis orthogonal to the inter‐slice direction:

(1) $$TTF_{i}\;\left(f_{i}\right)=\frac{\int TTF_{ij}\left(f_{i},f_{j}\right)df_{j}}{{\left.\int TTF_{ij}\left(f_{i},f_{j}\right)df_{j}\right|}_{f_{i}=0}}\;$$

where TTF_ij_ represents the 2D through‐plane TTF in the *i*‐*j* plane and TTF_i_ the inter‐slice TTF along the *i*‐direction, respectively.

The ratio of integrated area under TTF curves served as a task‐based metric for spatial resolution, offering a single‐number summary of performance across spatial frequencies. The following ratio based on the measured TTFs was used to compare the spatial resolution between R2 and R1 images:

(2) $$R_{TTF}=\frac{\int TTF_{R2}\left(f\right)df}{\int TTF_{R1}\left(f\right)df}\;$$

This ratio quantified the overall loss of task‐based resolution due to slice resampling in MPRs. A value close to 1.0 indicated close resolution between MPRs and axial images, while lower values indicated significant degradation. This ratio was calculated for each reconstruction of the three CT systems.

### 2.3 Noise power spectrum (NPS)

Three‐dimensional NPS of the R1 and R2 image stacks were measured from volumes of interest (VOIs) of 100 × 100 × 100 mm^3^ placed in the homogeneous volume of water at the center of the cubic phantom. The 3D NPS corresponds to the magnitude squared of the 3D Fourier Transform of each VOI, calculated using equation (3).

(3) $$\begin{array}{cc} & \mathrm{NPS}\;\left(f_{x},f_{y},f_{z}\right)=\frac{\Delta x\cdot \Delta y\cdot \Delta z}{N_{x}\cdot N_{y}\cdot N_{z}}\\ & {\left|\int \int \int \left(d\left(x,y,z\right)-\overset{-}{d}\right)\cdot \exp\left(-i2\pi \left(f_{x}x+f_{y}y+f_{z}z\right)\right)\mathrm{dxdy}\mathrm{dz}\right|}^{2} \end{array}$$

In equation (3), *d*(*x,y,z*) is the pixel value at the position (*x,y,z*), $\bar{d}$ is the mean pixel value in the VOI, *N_x_*, *N_y_*, *N_z_* and *Δx*, *Δy*, *Δz* are the number of voxels and voxel spacing in the x‐, y‐ and z‐directions, respectively. 3D NPS have the unit HU^2^mm^3^. No windowing nor detrending of uniform images were made for NPS calculation. 2D NPS maps were obtained by integrating the 3D NPS along the frequency axis perpendicular to the 2D NPS plane. 1D NPS curves are averages of 2D NPS over 360°, excluding the zero spatial frequencies of the NPS. 2D NPS maps and 1D NPS curves have the unit HU^2^mm^2^.

The following ratio based on the measured NPS was used to quantify the relative noise power present in R2 (MPRs) compared to R1 (axial) images:

(4) $$R_{NPS}=\frac{\int NPS_{R2}\left(f\right)df}{\int NPS_{R1}\left(f\right)df}\;$$

The integration was performed over all non‐zero spatial frequencies of the NPS. Values below 1.0 indicated noise reduction in MPRs, while values above 1.0 suggested amplification due to resampling or interpolation. The ratios were reported for each CT system, kernel, and slice setting.

The average NPS frequency, denoted *f_NPS_*, was used to quantify the fineness of noise texture of both R1 and R2 images:

(5) $$f_{NPS}=\frac{\int NPS\left(f\right)\cdot f\;df}{\int NPS\left(f\right)\;df}\;$$

### 2.4 Detectability index

A detectability index (*d’*) was calculated for spherical objects with diameters ranging from 0.5 mm to 5.0 mm, in both R1 (axial) slices and MPRs reconstructed under various conditions (kernel, slice thickness, overlap). We considered spheres of contrast *C *= 100 HU embedded in a homogeneous water volume. *d’* values were calculated using the non‐prewhitening with eye filter (NPWE) model observer. The NPWE detectability index was expressed in a 2D in‐plane “slice detectability” form by integrating 3D metrics in the NPWE model.^43^ Assuming the separability between the in‐plane and the orthogonal inter‐slice TTFs (both governed by independent parameters), the NPWE detectability index *d’* for the slice reconstructed in the *i*‐*j*‐plane was calculated using equation (6).

(6)

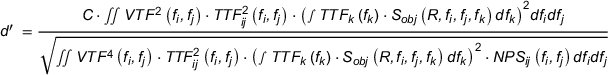




The Fourier spectrum $S_{obj}\left(R,f_{i},f_{j},f_{k}\right)$ of a sphere of radius *R* is given in equation (7), where J_3/2_ is the Bessel function of the first kind of order 3/2.

(7) $$\begin{array}{cc} S_{obj}\;\left(R,f_{i},f_{j},f_{k}\right) & =R^{3/2}\cdot {\left(f_{i}^{2}+f_{j}^{2}+f_{k}^{2}\right)}^{-3/4}\\ & \;\cdot J_{3/2}\left(2\pi R\sqrt{f_{i}^{2}+f_{j}^{2}+f_{k}^{2}}\right) \end{array}$$

The visual transfer function (VTF) of the human eye corresponds to an approximation of the Barten contrast sensitivity function of the human eye^44^ with a general form given in equation (8).^45^, ^46^, ^47^

(8) $$VTF\;\left(f_{i},f_{j}\right)=f^{n}\;\cdot exp\left(-cf^{a}\right)$$

Where $f={\left(f_{i}^{2}+f_{j}^{2}\right)}^{1/2}$ is the spatial frequency expressed in mm^−1^. In this study, the parameters *n*, *c* and *a* were chosen equal to 1.0, 2.2 mm^−1^ and 1.0, respectively, such that the maximum VTF response was around 4.0 mm^−1^.^43^, ^48^

## 3 RESULTS

### 3.1 Spatial resolution

**FIGURE 2:**
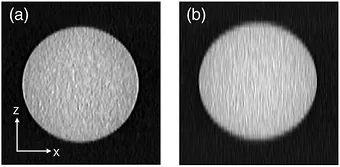
Anisotropic in‐plane resolution in MPRs due to the reconstruction parameters used for the R1 (axial) images. A) R1 slices “T” (Thin) and EE kernel. B) R1 slices “M” (Medium) and HR kernel.

**FIGURE 3:**
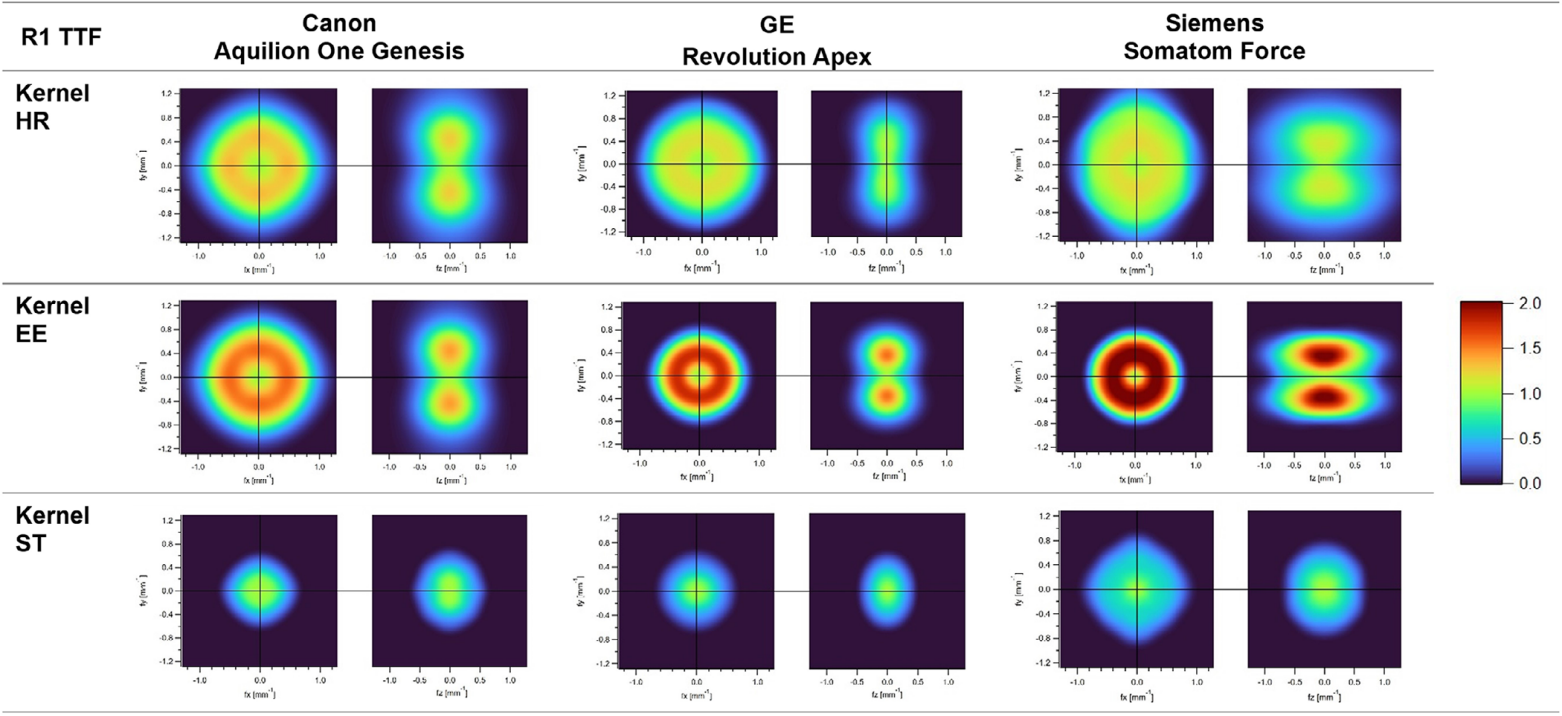
In‐plane TTF_xy_ and through‐plane TTF_yz_ of the R1 (axial) images for the three kernels (thin slices “T” with 50% overlap). Through‐plane TTF_xz_ (not shown) are symmetrical by 90° to TTF_yz_.

**FIGURE 4:**
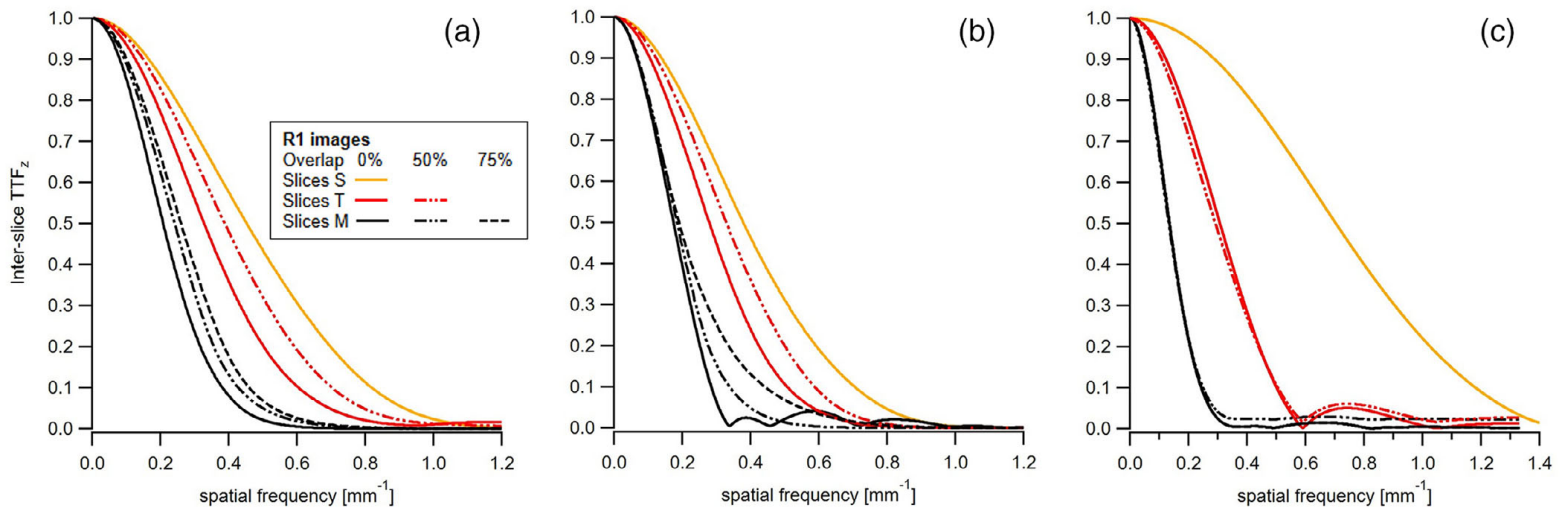
Inter‐slice TTF_z_ of the R1 (axial) images for three slice thicknesses (S = submillimeter, T = thin, M = medium slices) and intervals (overlaps 0%, 50% and 75%). The TTF_z_ were obtained by integrating the through‐plane TTF_yz_ of the R1 images in the y‐direction. A) Canon Acquilion One Genesis. B) GE Revolution Apex. C) Siemens Somatom Force.

The axial and longitudinal directions had very different spatial resolutions on the three CT systems. In‐plane resolution was globally isotropic on R1 slices but showed strong anisotropy between the axial xy‐ and longitudinal z‐directions on MPRs (Figure 2). In‐plane TTFs of R1 images were shaped by kernels (Figure 3), while inter‐slice TTFs (TTF_z_) were mainly determined by the R1 slice thickness and remained independent of kernels (Figure 4). The TTF_z_ improved primarily by reducing the R1 slice thickness and, to a lesser extent, with an increased overlap for Canon and GE systems. Axial and longitudinal resolutions are thus governed by different and independent reconstruction parameters.

**FIGURE 5:**
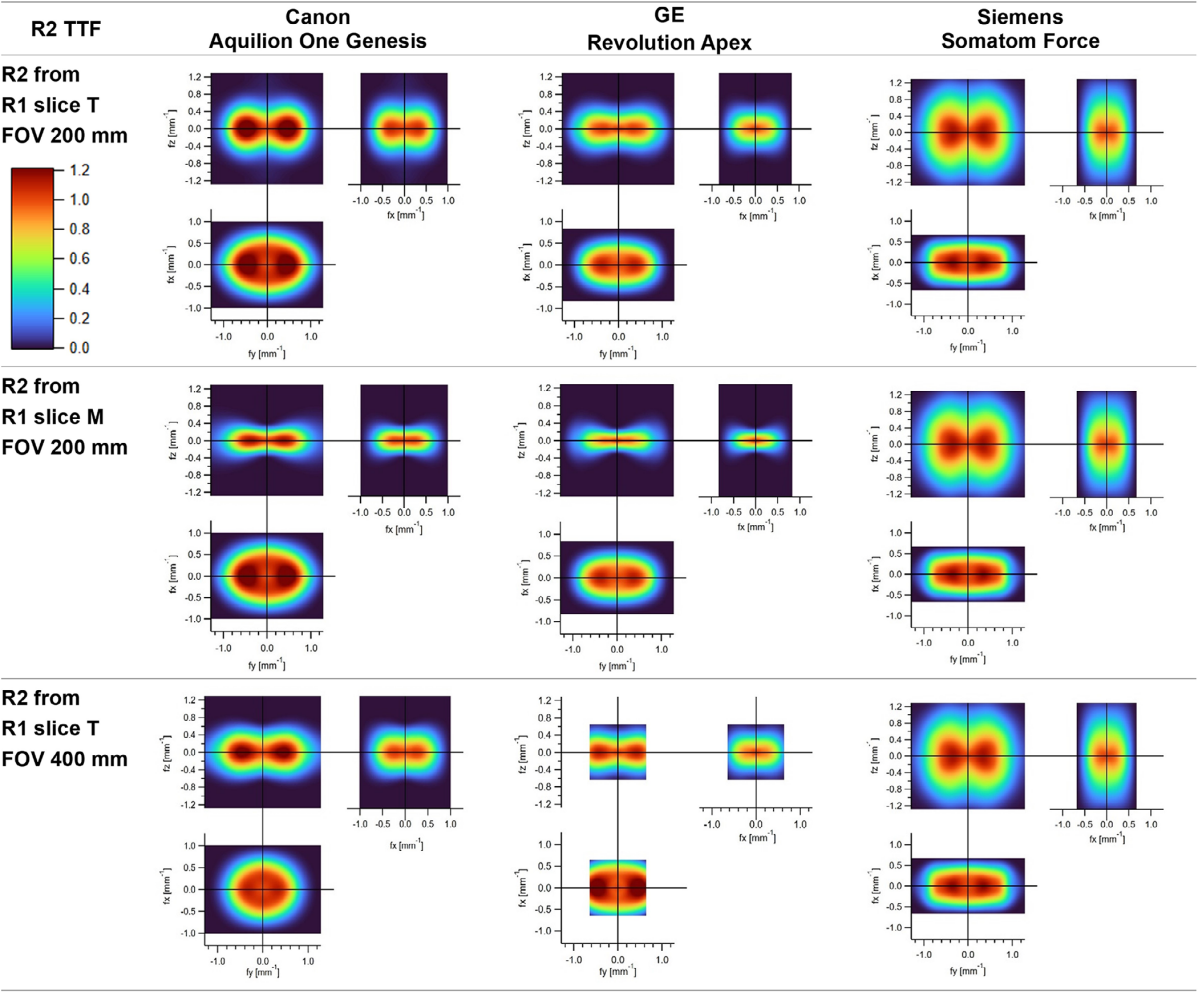
In‐plane TTF_yz_, and through‐plane TTF_xy_ and TTF_xz_ of the sagittal MPRs reconstructed from different R1 slice samplings with the kernel HR (T = thin, M = medium R1 slices).

The coronal and sagittal MPRs differed only by a permutation between the x‐ and y‐axes. We therefore arbitrarily chose to present the results of sagittal slices for MPRs. Unlike R1 images, the MPRs exhibited different resolutions along the x‐ and y‐axes due to the reslicing of the R1 volume used for their reconstruction. Consequently, the TTFs of MPRs differed in the three orthogonal planes, as shown in Figure 5. For both high‐resolution (HR) and edge‐enhancing (EE) kernels, MPRs showed high resolution and edge enhancement along the x‐ and y‐axes but a pronounced low‐pass behavior in the z‐axis. The MPRs kept additionally R1 voxel‐size resolution limits on the Canon and GE systems, resulting in degraded resolution, aliasing and partial volume effects when using thick R1 slices (Figure 5). The frequency cutoff and low‐pass crop due to the voxel size chosen for the R1 slices—R1 pixel size and slice thickness / interval—lead to reduced in‐plane spatial resolution of MPRs in the longitudinal z‐direction. The GE system did not allow to reconstruct MPRs with voxel sizes smaller than those of R1 images. On the contrary, the Siemens system used independent volume sampling for MPRs to decouple spatial resolution from primary reconstruction parameters (R1 pixel size, slice thickness, interval). This ensured consistent MPR image quality across varying R1 samplings.

**FIGURE 6:**
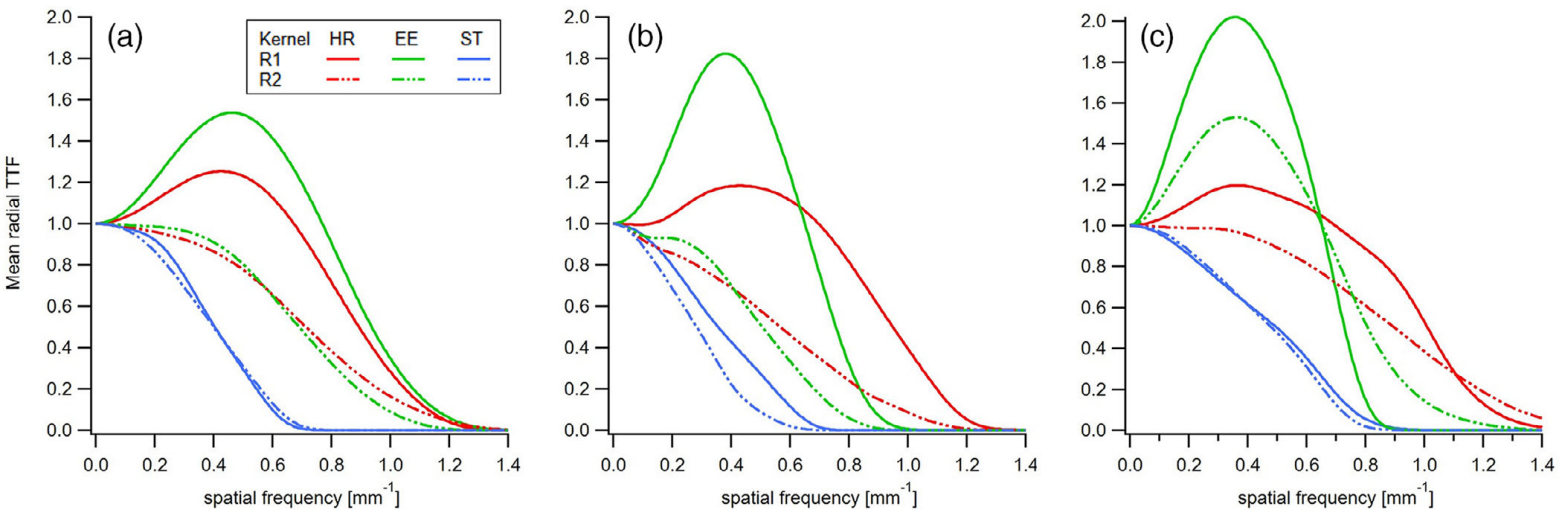
Comparison of the mean radial TTFs between the R1 (axial) images (thin slices “T” with 50% overlap) and the resulting MPRs for the kernels HR, EE and ST. a) Canon Acquilion One Genesis. b) GE Revolution Apex. c) Siemens Somatom Force.

Consequently, the overall in‐plane resolution of MPRs was lower than that of R1 slices, in particular for HR and EE kernels (Figure 6). While the HR or EE kernels introduced marked anisotropy, the ST kernels decreased the contribution of high frequencies to the axial plane, yielding more isotropic TTFs. Although these general trends were similar for all three CT systems, the Siemens system showed the smallest degradation in MPR resolution whereas GE showed the largest. For the HR kernel, the ratio R_TTF_ was equal to 0.826 for the Siemens, regardless of the R1 slice thickness / interval (Table 2). For the Canon, it was equal to 0.723 for S slices and decreased to 0.674 and 0.399 for T and M slices (without overlap), respectively. The GE gave the lowest R_TTF_, equal to 0.596, 0.467 and 0.295 for S, T and M slices without overlap, respectively. This ratio increased by 10% for a 50% overlap and by 15% for a 75% overlap compared to non‐overlapped R1 slices. The ratio R_TTF_ was higher and close to 1.0 for ST kernels, between 0.868 (GE) and 0.929 (Canon).

**TABLE 2:** Ratios R_TTF_ and R_NPS_ between MPRs and axial (R1) images, and average NPS frequency (f_NPS_ in mm^−1^) for different kernels and slice thicknesses / intervals (S = submillimeter, T = thin, M = medium R1 slices).

		Canon Aquilion One Genesis				GE Revolution Apex				Siemens Somatom Force			
		R_TTF_	R_NPS_	f_NPS_ R1	f_NPS_ R2	R_TTF_	R_NPS_	f_NPS_ R1	f_NPS_ R2	R_TTF_	R_NPS_	f_NPS_ R1	f_NPS_ R2
HR	S 0%	0.72	0.71	0.53	0.39	0.60	0.60	0.65	0.43	0.83	0.45	0.69	0.63
	T 0%	0.61	0.63	0.53	0.33	0.47	0.56	0.65	0.40	0.83	0.84	0.66	0.63
	T 50%	0.67	0.69	0.53	0.35	0.53	0.62	0.64	0.41	0.83	0.85	0.66	0.63
	M 0%	0.40	0.60	0.52	0.28	0.30	0.56	0.66	0.36	0.83	1.27	0.66	0.63
	M 50%	0.48	0.75	0.53	0.29	0.33	0.61	0.65	0.36	0.83	1.28	0.66	0.63
	M 75%	0.45	0.75	0.52	0.30	0.35	0.64	0.64	0.41	0.83	1.27	0.66	0.63
EE	T 50%	0.63	0.70	0.59	0.39	0.60	0.79	0.43	0.28	0.87	0.97	0.43	0.40
ST	T 50%	0.93	0.88	0.26	0.21	0.87	0.87	0.27	0.20	0.91	0.90	0.34	0.28

### 3.2 Noise power spectra (NPS)

**FIGURE 7:**
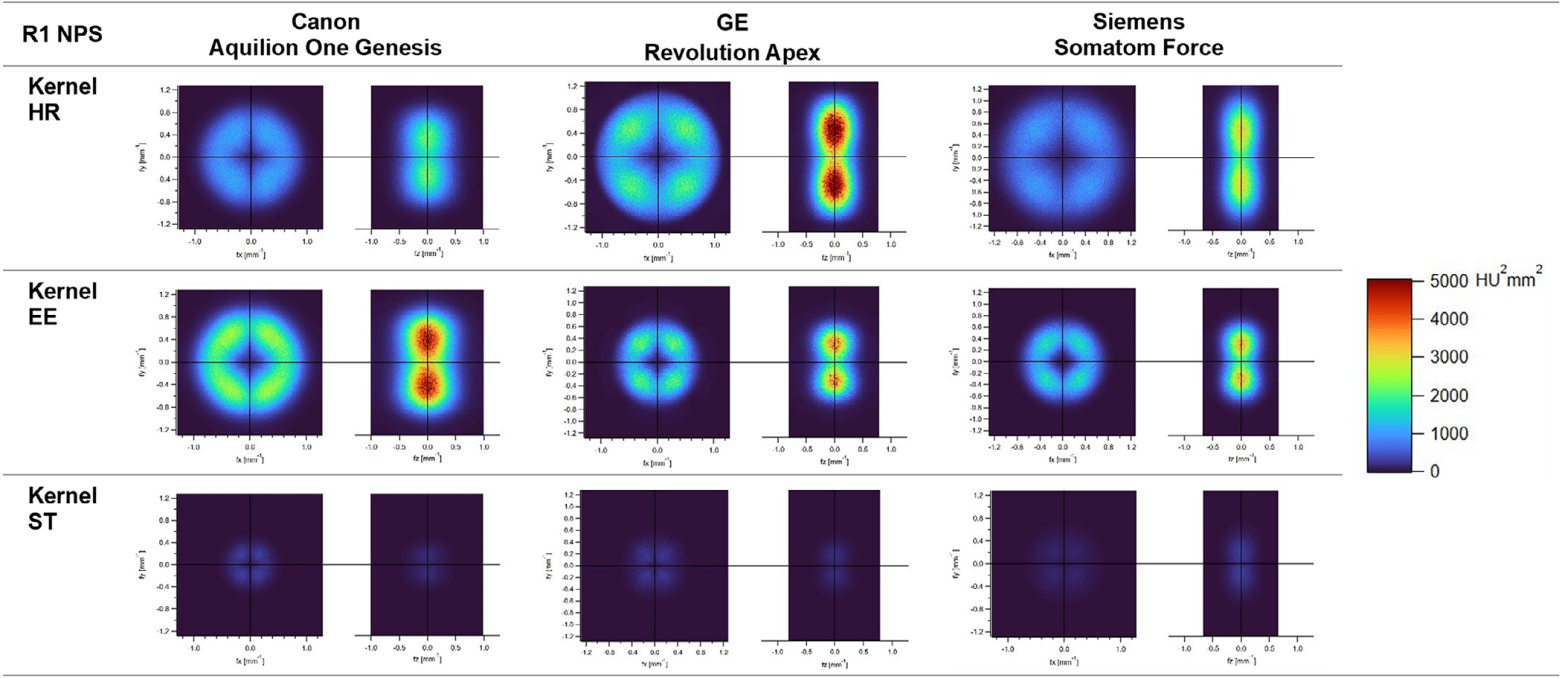
In‐plane NPS_xy_ and through‐plane NPS_yz_ of the R1 (axial) images for the three kernels (thin slices “T” with 50% overlap). Through‐plane NPS_xz_ (not shown) are symmetrical by 90° to NPS_yz_.

**FIGURE 8:**
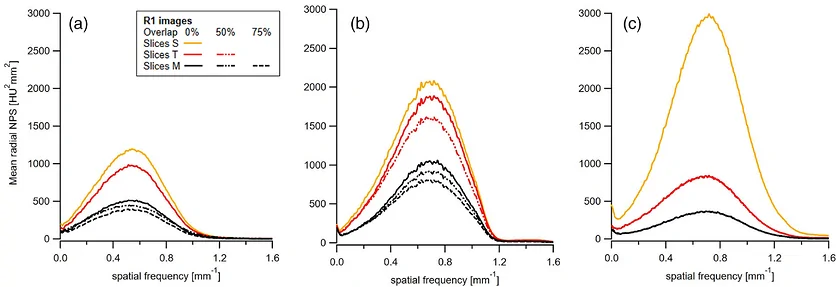
Mean radial NPS of R1 (axial) images for different slice thicknesses / intervals (S = submillimeter, T = thin, M = medium R1 slices). a) Canon Acquilion One Genesis. b) GE Revolution Apex. c) Siemens Somatom Force.

Following the differences in spatial resolution, the NPS differed strongly between the axial and longitudinal directions for the three CT systems. The reconstruction kernel was the main factor influencing the axial NPS whereas the NPS in the z‐axis was mainly determined by the R1 axial sampling. The three kernels demonstrated different trade‐offs between image noise and spatial resolution in the axial xy‐plane (Figure 7). ST kernels transmitted only low‐frequency noise whereas sharp HR and EE kernels produced greater high‐frequency noise. In‐plane NPS of R1 images was isotropic, increasing for thicker slices and, for Canon and GE systems, for decreased overlap (Figure 8). Conversely, the NPS of the Siemens images showed no change for the different R1 slice overlaps tested in this study.

**FIGURE 9:**
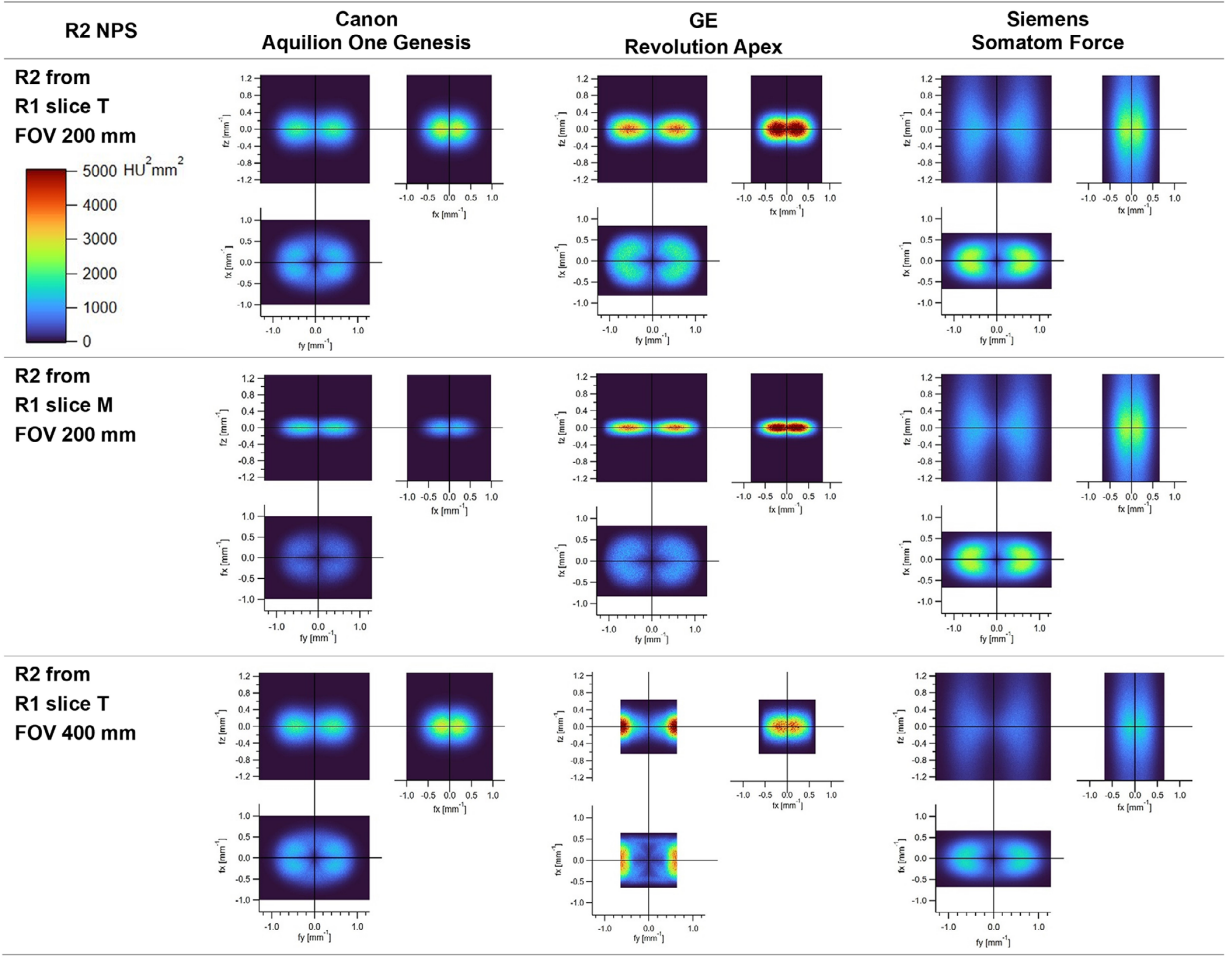
In‐plane NPS_yz_, and through‐plane NPS_xy_ and NPS_xz_ of the sagittal MPRs reconstructed from different R1 slice samplings with the kernel HR (T = thin, M = medium R1 slices).

The NPS of MPRs was highly isotropic, with mid‐pass characteristics in the axial direction and a low‐pass shape in the longitudinal direction (Figure 9). The NPS anisotropy between the three orthogonal planes followed the same trends as those described for the TTF. For both Canon and GE systems, the primary slice thickness / interval chosen to reconstruct the R1 slices influenced the magnitude, shape and bandwidth of the NPS in the z‐axis, with possible noise aliasing.

**FIGURE 10:**
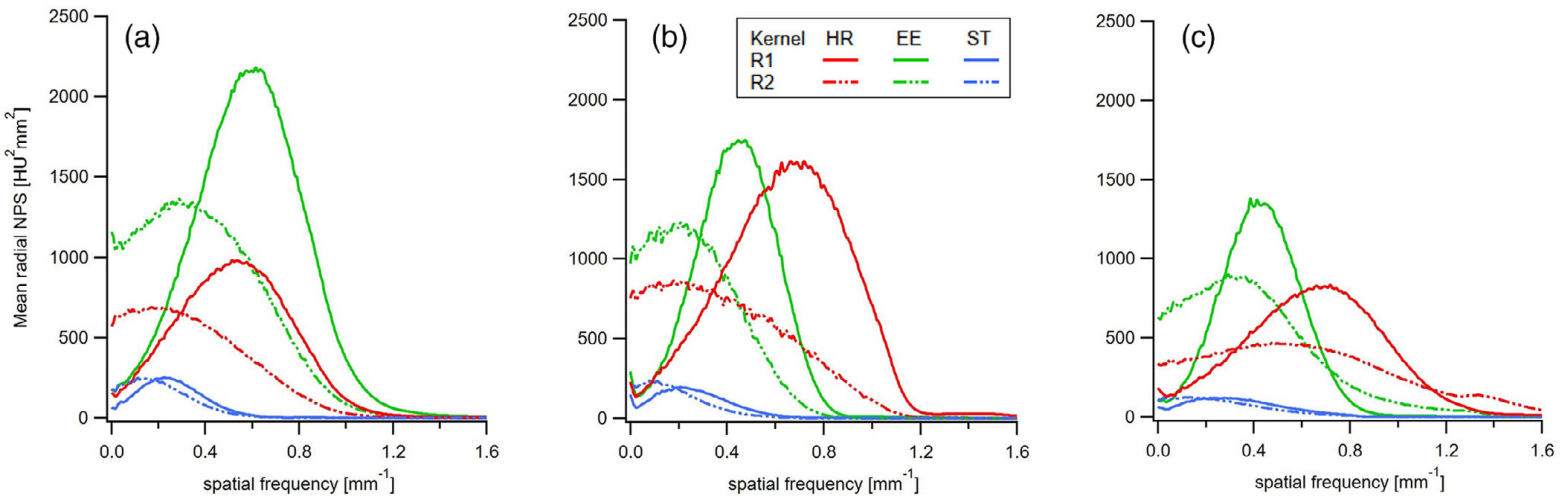
Comparison of the mean radial NPS between the R1 (axial) images (thin slices “T” with 50% overlap) and the resulting MPRs for the kernels HR, EE and ST. a) Canon Acquilion One Genesis. b) GE Revolution Apex. c) Siemens Somatom Force.

As shown in Figure 10, the average in‐plane noise magnitude and texture differed strongly between the R1 slices and MPRs for the HR and EE kernels. ST kernels rolled off the contribution of high frequencies to the axial plane and gave more isotropic NPS. For the HR kernel, the ratio R_NPS_ ranged from 0.453 to 1.276 for the Siemens, which showed the highest range of noise differences between MPRs and R1 images for the different R1 slice thicknesses / intervals (Table 2). This ratio varied less for the Canon and GE systems. For the Canon, it was equal to 0.714, 0.631 and 0603 for the S, T and M slices (without overlap), respectively. The GE gave the lowest range, with ratios equal to 0.598, 0.561 and 0.560 for S, T and M slices without overlap, respectively. This ratio increased by 10 ‐ 20% for 50% overlap and did not evolve significantly (less than 4%) for an overlap of 75%. Although strongly anisotropic, the noise texture was in overall of lower frequency on the MPRs than that on the R1 images, as evidenced by the decrease in the f_NPS_ parameter (Table 2) due to the lower resolution in the longitudinal direction.

### 3.3 Detectability index

**FIGURE 11:**
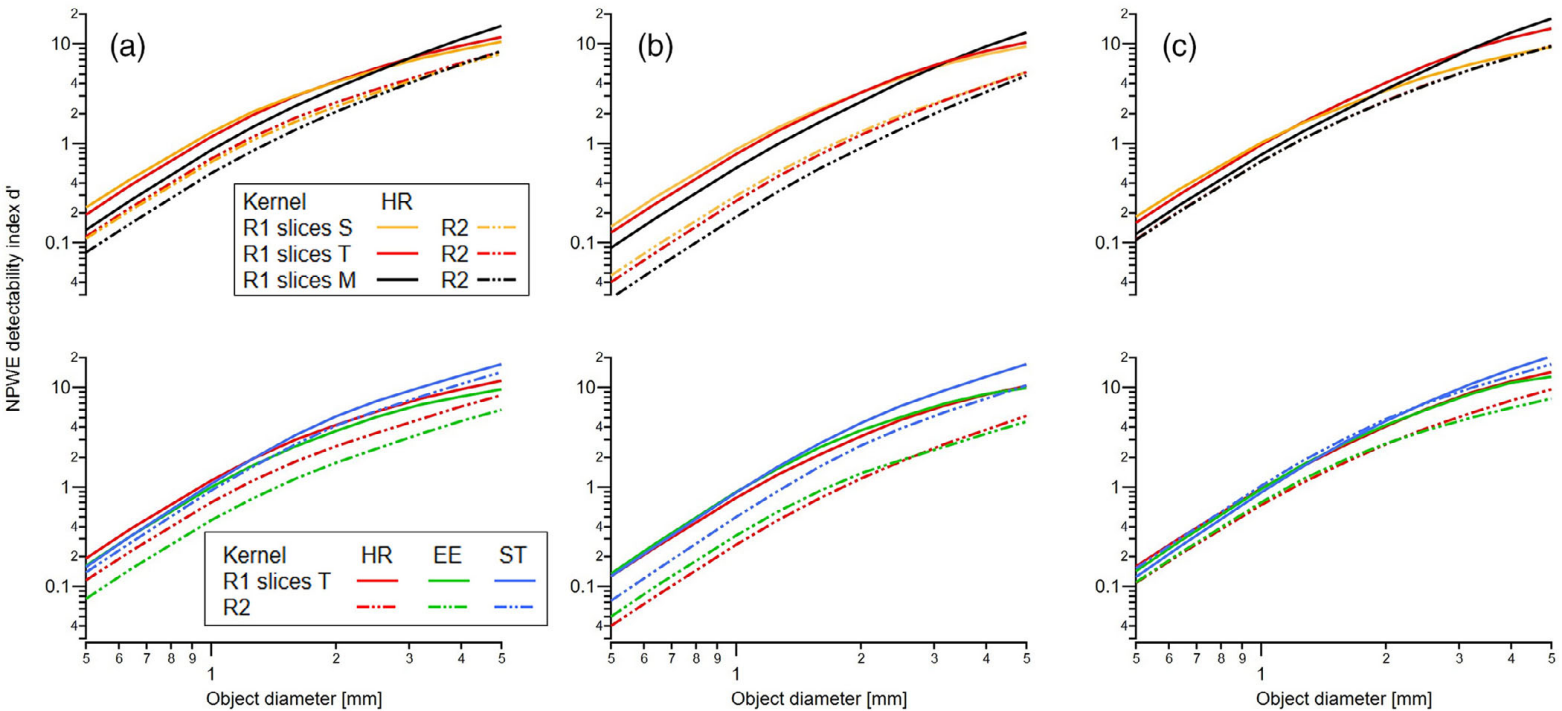
NPWE detectability index *d’* of R1 (axial) images and R2 (MPRs) for different R1 reconstruction parameters (R1 slice thickness: S = submillimeter, T = thin, M = medium). a) Canon Acquilion One Genesis. b) GE Revolution Apex. c) Siemens Somatom Force.

The thinnest R1 (axial) slices yielded the highest index *d′* for small objects, with the optimal slice thickness increasing progressively with object size (Figure 11). For the Canon, for example, submillimetre (S) slices outperformed thin (T) slices for objects smaller than ≈ 4 mm, while medium‐thickness (M) slices became optimal for the largest objects studied (5 mm) (Figure 11a). This behavior reflects the tradeoff between longitudinal resolution and noise, determining the optimal thickness as a function of object size.

As expected from the resolution loss quantified by R_TTF_, the MPRs had lower detectability than the R1 slices for all object sizes studied (0.5 to 5.0 mm). For the Canon and GE systems, the size‐dependent behavior observed for the R1 slices propagated to the MPRs, since the longitudinal sampling of the primary slices was carried over to the MPRs. MPRs reconstructed from thin R1 slices retained higher detectability for small objects than those reconstructed from thick R1 slices. This propagation was absent for the Siemens system, whose independent volumetric resampling decoupled MPR detectability from the R1 slice thickness.

Smooth (ST) kernels provided more isotropic image quality and the highest *d′* in MPRs for all three systems, compared to HR and EE kernels. Overall, the Siemens system maintained the best detectability in MPRs and the GE system showed the greatest decrease between R1 slices and MPRs.

## 4 DISCUSSION

This study quantitatively evaluated the image quality of primary axial images and MPRs on three recent CT systems. A dedicated cubic phantom was designed to measure spatial resolution and noise in the same conditions in the axial, coronal and sagittal planes. Acquisition parameters (kV, mAs, pitch) and reconstruction parameters (kernels, pixel size, slice thickness) were matched as closely as possible across the three CT systems to ensure comparability. Beyond simple variations in acquisition and reconstruction parameters, the results highlighted intrinsic differences in spatial resolution and noise between the axial and longitudinal directions. For all three CT systems, the parameters used to reconstruct the R1 (axial) slices (reconstruction algorithm, kernel, filter) produced a trade‐off between spatial resolution and noise in the axial plane, which was carried over into the axial direction of MPRs. In particular, the HR and EE kernels improved spatial resolution and edge visibility compared to ST kernels but increased noise in the x‐ and y‐axes, while the z‐axis had reduced spatial resolution and noise. These results confirm that the in‐plane resolution of MPRs in a specific direction can be determined by an angular weighting between the in‐plane and longitudinal spatial resolutions.^32^ For the Canon and GE systems, the longitudinal sampling (slice thickness and interval) chosen for the primary axial (R1) images was carried over to MPRs, which could result in poor resolution and a highly anisotropic quality if thick R1 slices were chosen for the primary reconstruction. In contrast, the Siemens system applied a volumetric sampling to MPRs independent of the R1 voxel size, thus preserving a higher resolution in the MPRs.

In our study, detectability was assessed using the NPWE model, which has previously shown good agreement with human observer performance in previous CT and CBCT studies.^43^, ^48^, ^49^, ^50^ All three CT systems involved in this study showed reduced detectability in MPRs compared to R1 images. This loss was system‐ and reconstruction parameters‐dependent. The Siemens system performed best, with the lowest decrease and the highest detectability index in MPRs for all three kernels. The Canon and GE systems, which kept R1 sampling on MPRs, showed a decline in MPR detectability with increasing R1 slice thickness, underscoring the need to reconstruct MPRs from the thinnest possible R1 slices. For the Canon and GE systems, using an R1 slice interval equal to half the slice thickness increased the spatial resolution in the z‐axis and reduced the noise level. On both systems, oversampling reduced noise by effectively averaging out random fluctuations in the data. It is important to note, however, that there is an optimal slice thickness for any given imaging scenario, depending on the size and nature of the structures being imaged and the amount of noise acceptable.^51^, ^52^

Our results showed that the MPRs of the three CT systems were reconstructed from the processed R1 image stack, and not from the raw sinograms, as the R1 kernels and filtering features were still present in the axial directions of the MPRs. For the Siemens system, the MPRs were reconstructed from finely resampled R1 volumes in the axial direction, unlike the Canon and GE systems. The image quality of MPRs is therefore strongly constrained by the reconstruction parameters used for R1 slices. It is therefore advisable for the Canon and GE systems to reconstruct MPRs by selecting thin R1 slices with at least 50% overlap to minimize the image quality loss and then choosing the optimal MPR slice thickness. Submillimeter (S) slices are not necessarily recommended, as the gain in resolution compared to thin (T) slices can be much less than the increase in noise level. Applying at least 50% overlap for R1 images improved the longitudinal resolution and decreased the noise level in the resulting MPRs. For applications requiring isotropic reconstruction or emphasizing low‐contrast detectability, smooth reconstruction kernels should be selected. Choosing a smooth standard reconstruction kernel is an effective method for obtaining isotropic 3D volumes, more efficient than, for example, trying to match the slice widths of R1 and R2 images, which is not always feasible anyway. There is, however, no universal kernel choice: for MPRs, smooth kernels are preferable when isotropic resolution and low noise are essential, while sharp kernels may be justified for applications requiring high‐frequency details in the axial plane (e.g. coronary stents, lung nodules, bone fractures), albeit with reduced MPR performance. For clinical applications where sharp kernels significantly impact diagnostic accuracy, MPRs can give reduced detectability than R1 images. This could be the case, for example, for the assessment of calcifications and coronary stents in cardiac CT^53^ or on the detection of lung nodules in low‐dose CT.^54^

The scope of this study was limited on three recent CT systems with adaptive statistical iterative reconstructions commonly used for chest CT, abdominal CT, and other clinical applications. Other reconstruction algorithms and kernels than the three chosen in this study would have resulted in different resolution‐noise trade‐offs. Additional protocols, particularly those involving AI‐based reconstructions, may exhibit different resolution–noise characteristics. It is also important to note that the cubic test‐object used to measure physical image quality parameters was not designed for image quality assessment with anthropomorphic structures, which might be necessary for evaluating advanced AI‐based reconstruction algorithms. Therefore, adaptive image processing might yield different results when applied to test‐objects with inhomogeneous backgrounds. Further research using test‐objects with anthropomorphic structured background or targets is needed to investigate the extent to which our results could be generalized to more sophisticated reconstruction techniques. However, while MPR images are incredibly useful for visualizing anatomy, they can sometimes suffer from poorer image quality compared to the R1 images, especially regarding sharpness and the display of fine details.^39^, ^55^ This can be due to factors like the interpolation methods used in creating the MPR images, or the way noise and artifacts are handled during the reconstruction process. Advancements in imaging technology and reconstruction algorithms are constantly improving MPR image quality.^56^, ^57^ For instance, the use of thinner slices and more sophisticated reconstruction techniques may help improve longitudinal resolution and alleviate some of these limitations.

## 5 CONCLUSIONS

This work introduced a novel method for assessing the three‐dimensional image quality of CT scanners encompassing both primary axial images and MPRs. The results highlighted the highly anisotropic and inferior image quality of MPRs compared to axial images, mainly due to lower resolution in the longitudinal axis. The image quality of MPRs is primarily driven by the selected reconstruction kernel in the x‐ and y‐axes and a pronounced low‐pass behavior in the z‐axis. Resolution limits due to primary reconstruction sampling can moreover be reported in MPRs for some CT systems, leading to degraded resolution, aliasing and partial volume effects when using thick R1 slices. Although MPRs can improve the visualization of structures in different planes, the spatial resolution inherent to the original CT volume may not be perfectly preserved in the MPRs, especially when thick axial slices are used for primary reconstruction. To optimize task‐based image quality, MPRs should systematically be reconstructed from a thin axial slice thickness with a minimum overlap of 50%. Furthermore, smooth kernels provide more isotropic image quality and offer the highest detectability, making them the preferred choice when overall MPR quality is prioritized.

## AUTHOR CONTRIBUTIONS

• Pascal Monnin: conceptualization, design of the test object, acquisition, image analysis, interpretation of the work, writing the manuscript, review & editing, and final approval of the manuscript.

• Damien Racine: conceptualization, design of the test object, acquisition, interpretation of the work, contribution to the critical review and final approval of the manuscript.

• Anaïs Viry: conceptualization, design and interpretation of the study, contribution to the critical review of the manuscript

• Fabio Becce: conceptualization, design and interpretation of the study, contribution to the critical review of the manuscript

## CONFLICT OF INTEREST STATEMENT

The authors declare no conflicts of interest.

## ETHICAL APPROVAL STATEMENT

The authors have nothing to report.

## Data Availability

The data that support the findings of this study are available from the corresponding author upon reasonable request.
